# Supplementary figures and images for: The spindle assembly checkpoint and the spatial activation of Polo kinase determine the duration of cell division and prevent tumor formation
Source: PLoS Genet. 2022 Apr 4;18(4):e1010145. doi: 10.1371/journal.pgen.1010145 (PMC9009772; doi:10.1371/journal.pgen.1010145)

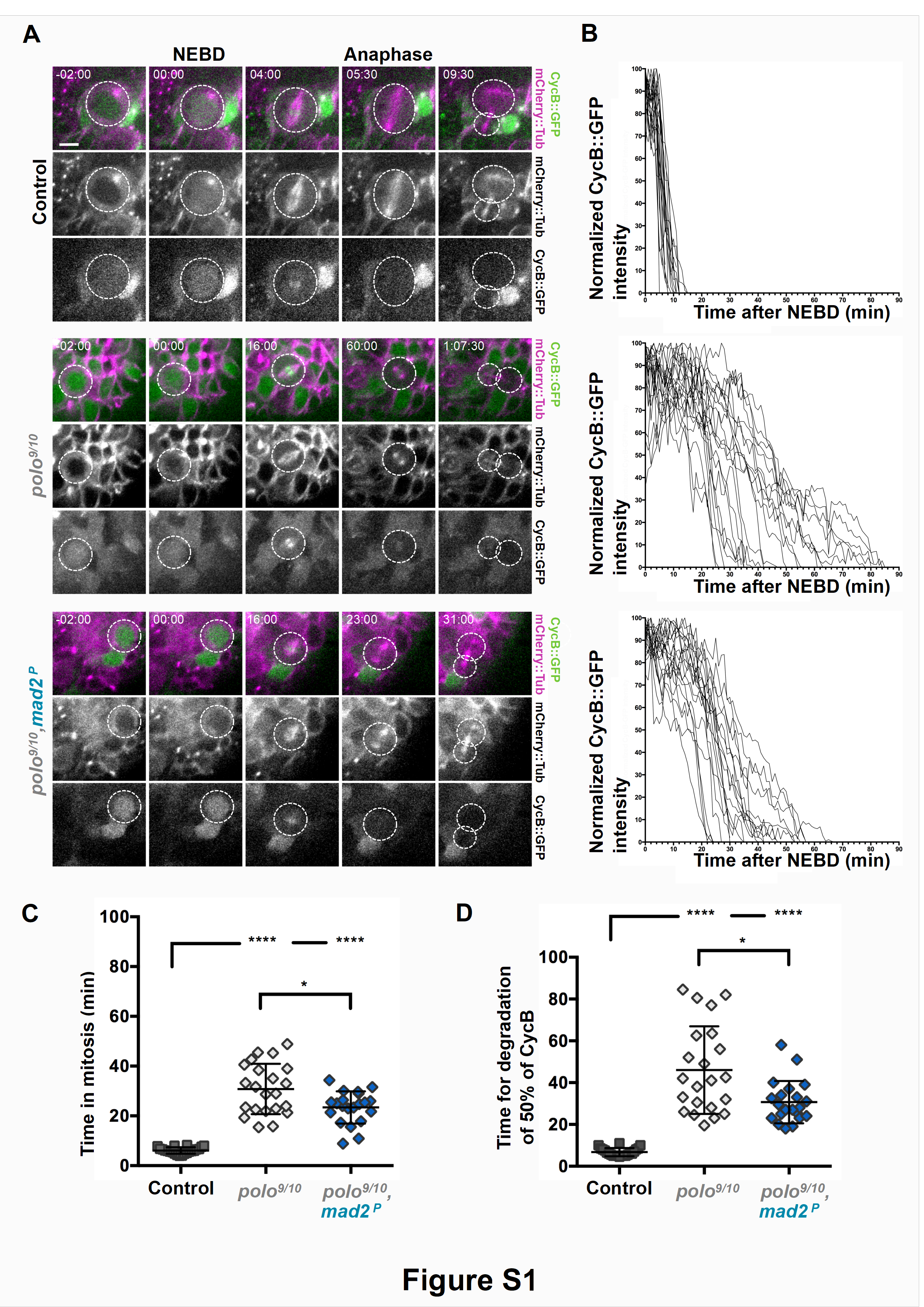

Supplement: S1 Fig — A) Time-lapse imaging of mitosis in polo mutants in the absence or presence of Mad2. Selected image series of dividing NSCs of the indicated genotypes expressing mCherry::Tubulin (purple in the top panels and in the middle monochrome panels), and CyclinB::GFP (green and lower monochrome panels). The white dashed line outlines the dividing NSCs. Scale bar: 5 μm. Time is min:s (t = 00:00 is NEBD). B) Cyclin B::GFP degradation profiles in WT, in polo9/polo10 NSCs, and in polo9/polo10, mad2P double-mutant NSCs. C) Quantification of the time in mitosis (min) in NSCs for the indicated genotypes. D) Quantification of the time (min) required for 50% of Cyclin B::GFP degradation for the indicated genotypes. Mann-Whitney unpaired tests: ns: p>0.05; *: p<0.05; ****: p<0.0001. (TIF) [file pgen.1010145.s001.tif]

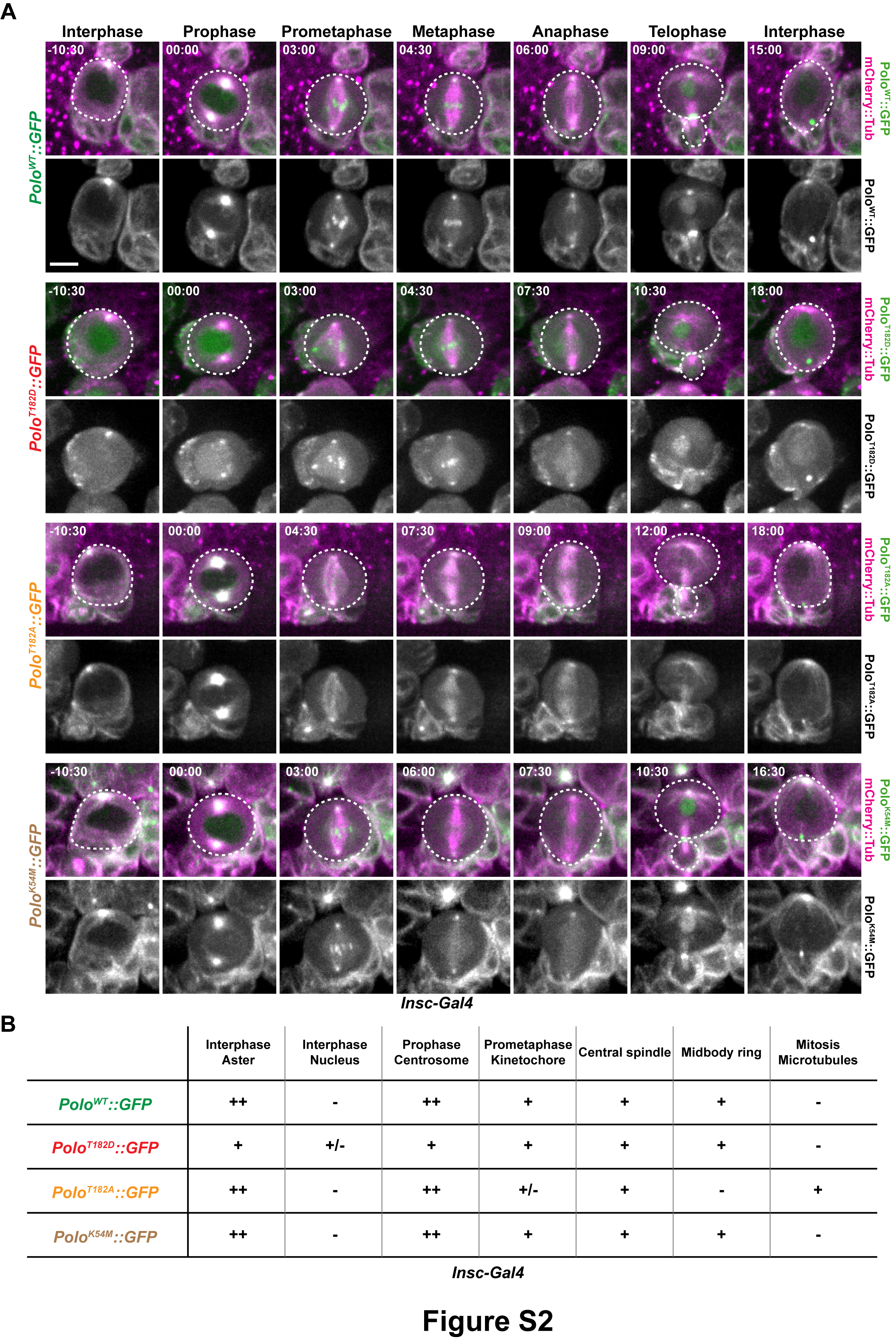

Supplement: S2 Fig — A) Time-lapse imaging of cell division in NSCs expressing the indicated Polo variants and mCherry::Tubulin. The white dashed line outlines the NSCs. Scale bar: 5 μm. Time is min:s (t = 00:00 is NEBD). B) Summary table of Polo variants localization patterns along cell cycle stages. The symbols “-”, “+/-”, “+”and “++” reflect absence, variable localization, moderate localization and strong enrichment, respectively. (TIF) [file pgen.1010145.s002.tif]

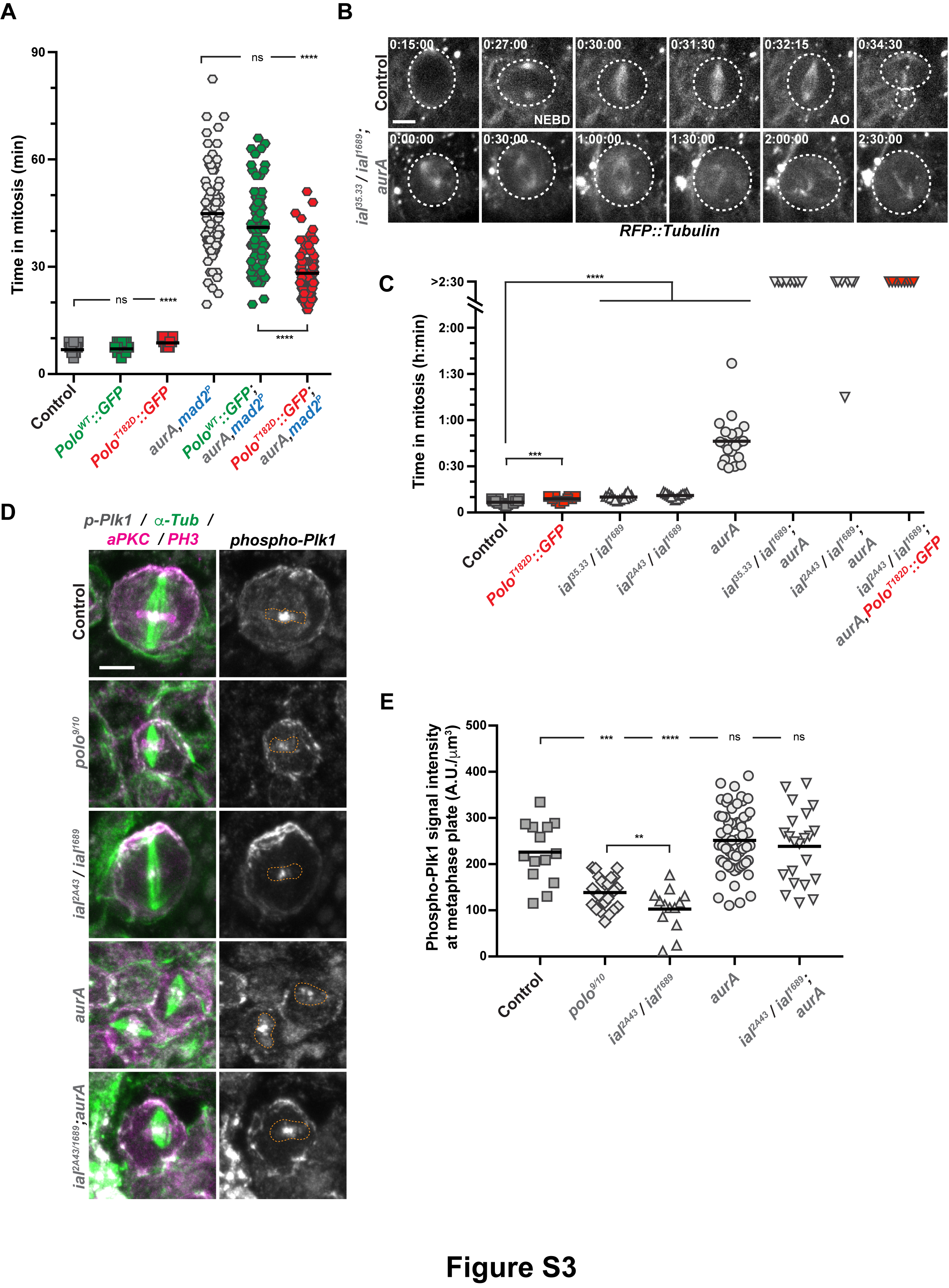

Supplement: S3 Fig — A) Quantification of the time in mitosis for the indicated genotypes. B) Time-lapse imaging of dividing NSCs for control and ial;aurA double mutant NSCs expressing RFP::Tubulin. The white dashed lines outline the dividing NSCs. Scale bar: 5 μm. Time is h:min:s (t = 0:00:00 is the beginning of the experiment). C) Quantification of the time in mitosis of NSCs of the indicated genotypes. The ial;aurA double mutant NSCs displayed a severe mitotic delay that could not be quantified since the vast majority did not complete mitosis during the duration of the experiment (2.5 hours). Mann-Whitney unpaired test: ns: p>0.05; ****: p<0.0001. D) Representative images of metaphase NSCs of the indicated genotypes stained for phospho-Plk1 (white in the merge), aPKC and Phospho-Histone H3 (magenta in the merge) and tubulin (green in the merge). The orange shapes in the right column outline the metaphase plates, according to the Phospho-Histone H3-labeled metaphase plate, in which phospho-Plk1 signal was measured. Scale bar: 5 μm. E) Quantification of phospho-Plk1 signal for the indicated genotypes, normalized by the area of measurement. Unpaired t-test: ns: p>0.05; **: p<0.01; ***: p<0.001; ****: p<0.0001. (TIF) [file pgen.1010145.s003.tif]

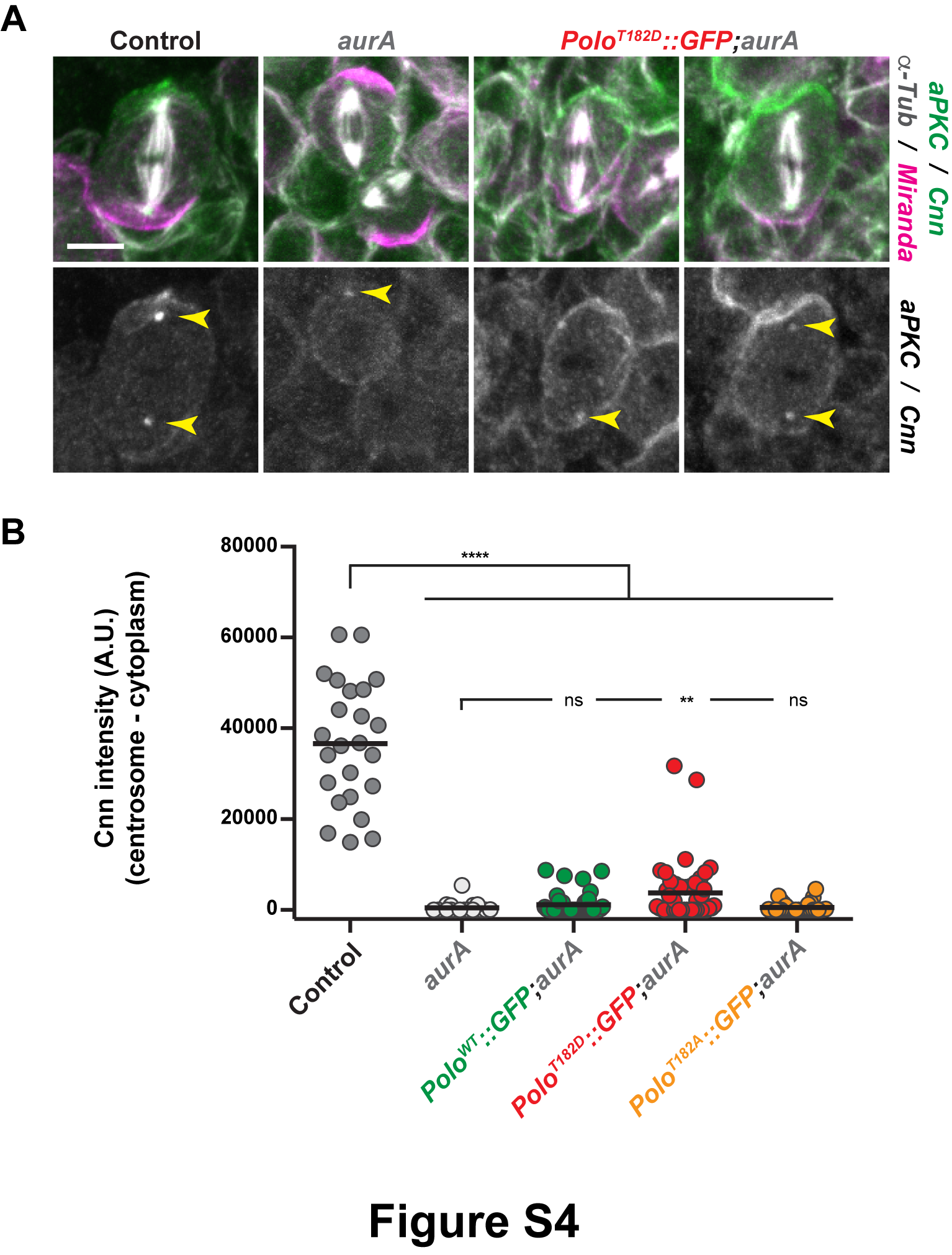

Supplement: S4 Fig — A) Representative images of metaphase NSCs of the indicated genotypes stained for Miranda (magenta in the merge), aPKC and Cnn (green in the merge) and tubulin (white in the merge). The yellow arrowheads in the bottom line highlight the Cnn-labeled spindle poles. Scale bar: 5 μm. B) Quantification of normalized Cnn intensity at spindle poles for the indicated genotypes. Mann-Whitney unpaired tests: ns: non-significant, *: p<0.05; **: p<0.01; ****: p<0.0001. (TIF) [file pgen.1010145.s004.tif]
