# Supplementary material for: The spindle assembly checkpoint and the spatial activation of Polo kinase determine the duration of cell division and prevent tumor formation
Source: PLoS Genet. 2022 Apr 4;18(4):e1010145. doi: 10.1371/journal.pgen.1010145 (PMC9009772; doi:10.1371/journal.pgen.1010145)
Supplement: Attachment — Submitted filename: plos genetics response to reviewers_25-02-22_EG.docx [file pgen.1010145.s009.docx]

------------------------------------------------------------------------------ 
Reviewer #1 (Evidence, reproducibility and clarity (Required)): 

**Summary:** 

This study focuses on the role of Polo kinase, a critical regulator of mitotic progression, in the control of asymmetric division in Drosophila neuroblasts. The main conclusion is that Polo acts downstream of AurA in the polarization neuroblasts and in the control of neuroblast amplification. The data feature primarily genetic manipulations coupled with both live imaging and cytological analyses. The authors show that previously published aurA mutant defects in neuroblasts can be suppressed by expression of an allele of Polo with an activation loop phosphomimetic mutation. Given that this site is a well-characterized and concserved AurA phosphorylation site on Polo, these data are taken as genetic evidence that Polo acts downstream of AurA in the fly NB tumor suppression pathway. The study also shows that, like aurA mutants, both neuroblast amplification and the mitotic lengthening are independent of the spindle assembly checkpoint (SAC) in polo mutants. 

**Major comments:** 

Figure 5 describes the suppression of aurA polarity, spindle alignment and overgrowth defects by PoloT182D expression. The Polo and PoloT182A alleles, which the authors' model predict will not suppress aurA defects, should be included in this analysis. Given that these transgenes have already been generated, obtaining these data should be quite feasible.

We have quantified the neuroblast numbers, polarity analyses, spindle orientation and centrosomal recruitment of Centrosomin in *aurA* mutant cells also expressing the WT and the TA variant. All analyses are now included in two modified figures.

-Figure 5 (Panels B, D, H and J)

-Figure S4 B (former figure S3).

The discussion and conclusions related to threshold effects of Polo activity are confusing and not supported. Expression of either inactivate alleles (T182A or K54M) or activated polo (T182D) slightly increases the length in mitosis in a wildtype background. The mechanistic basis for this phenotype is unclear, but seems unlikely to be due to an "excess of Polo activity caused by expression of PoloT182D in WT" (Page 7, line 1).

Expression of a PoloT182D mutant is known to generate a mitotic delay (Barbosa et al., 2020; Beck et al., 2013; Foley et al., 2011; Liu et al., 2012; Paschal et al., 2012). The “excess of kinase activity” is indeed inappropriate. We propose to change into “expression of a constitutively active Polo mutant”.

The inactive Polo kinases (PoloK54M and the PoloT182A mutants) have already been described to generate dominant negative effects that are mediated trough the Polo boxes (for review, Archambault et al., 2015 Bruisma et al., 2015). We have modified the text where needed.

**Minor Comments:** 

Cyclin B degradation was not monitored in this study, so statements such as "this reveals Polo thresholds of activation are essential for timely Cyclin B degradation... in vivo" should be toned down or removed. While this is a reasonable speculation, the supporting data are not provided.

We agree with this comment. We therefore have added Cyclin B-GFP degradation dynamics for the strong *polo* hypomorphic mutant associated with a loss of the SAC. This new experiment is now added in a new supplementary Figure S1. We now unambiguously correlate that a defective Cyclin B degradation is associated with a SAC-independent function of the Polo kinase.

There are a number of studies supporting a direct role for PLK1 in cell polarization outside of fly neuroblasts (for example, in the worm embryo), and it would be helpful to reference these studies in the discussion.

The appropriate references and reviews are now added in the revised version (From Gotta, Pintard, Goehring and Goldstein labs)

Reviewer #1 (Significance (Required)): 

This manuscript builds upon a previous study from the Giet lab showing in aurA mutants, NB display mitotic delay and neuroblasts overproliferation, and that these phenotypes are independent of the SAC (Caous 2015). Given that PLK1 is a well characterized AurA kinase and previously implicated in neuroblast asymmetric division, it is not particularly surprising that is Polo is acting downstream of AurA in neuroblasts. The significance of the study is limited to the extent that the mechanisms by which Polo acts in remains largely unaddressed. Nonetheless, given the relevance to the study of cell polarity, asymmetric division and tumorigenesis, there is likely to be broad interest in this study.

Indeed, we feel that our study will attract broad interest in the field and will be abundantly cited for years to come.

My expertise is in asymmetric cell division and cell polarity, and I do not study Drosophila. 

Reviewer #2 (Evidence, reproducibility and clarity (Required)): 

In their manuscript, Gallaud et al. propose that Polo and Aurora kinases cooperate during the asymmetric division of Drosophila neural stem cells (NSCs) to coordinate cell cycle progression and polarization in order to maintain a proper tissue homeostasis. Specifically, the authors suggest that phosphorylation by Aurora A at the spindle poles and by Aurora B at centromeres regulates Polo kinase activity levels, which subsequently determine cell division length independently of the spindle assembly checkpoint (SAC). Despite this dual regulation of Polo by Aurora A and B, Gallaud et al. further show that the main event that ensures NSC polarization and prevents brain tumor progression together with the SAC is the Aurora A-dependent activation of Polo. 

The conclusions drawn by the authors provide interesting new insights into how mitotic progression is coupled with the generation of asymmetry during cell division. However, and although the manuscript is of potential interest and may represent a significant advance in the knowledge in its field, it would definitely require to more strongly substantiate some of the authors' claims that are lacking of enough experimental support. I am especially concerned about the fact that a main body of results relies on the measurement of the length of mitosis using a methodology that might not be the most appropriate according to the nature of the mutants under study. Together with this, my main concerns regarding the manuscript are: 

1.- In the live-imaging experiments to evaluate the time in mitosis, it should be more clearly established how nuclear envelope breakdown and anaphase onset was determined. In the Methods section, the authors only cite Métivier et al. (2020). In this reference, mitotic entry was estimated by an increase of mCherry-tagged tubulin signal in the nuclear region that reflects dismantlement and permeabilization of the nuclear envelope to tubulin, while anaphase onset was determined as the first spindle elongation time point. This is also difficult to evaluate in the manuscript, since the authors only show representative images of the live-imaging microscopy experiments. Making the time-lapse movies for the same experiments further available to the readers would provide extra information and be more helpful to properly assess the extent of the delay.

This is a valid concern, and movies are now provided in this revised version.

- 2- In any case, an estimation of the time in mitosis based on mCherry-Tubulin signal, and thus on centrosome and spindle dynamics, might not be the best choice in the case of Polo mutants, due to the known role of this kinase in the regulation of the aforementioned processes. This could explain the wide fluctuation of the data obtained in the polo9/10 mutant background (e.g., Figure 1A). The same is true for Aurora A and B mutants, since these kinases also play important roles in centrosome and spindle regulation. A more careful determination of the time in mitosis by introducing additional cell cycle markers (e.g., Cyclin B-Cdk1, etc.) would be therefore highly recommended for these experiments.

It is standard in the field to measure the duration of mitosis as the time elapsed between Nuclear Envelope Breakdown (NEB) and Anaphase onset. These are discrete, punctual events that mark the beginning and the end of mitosis, respectively. The time of NEB is determined as the time when mCherry-Tubulin (cytoplasmic in interphase) becomes visible in the nuclear area. This signal largely corresponds to free Tubulin and is therefore not impacted by any alterations of centrosome or spindle functions in *polo* or *aurora* mutants. Therefore, we do not think an additional marker is necessary for our results to be valid. Anaphase onset, marked by the degradation of Cyclin B and the subsequent segregation of sister chromatins, is very well visible because the shadow of the chromosomal mass, visible at the spindle equator, suddenly extends and is easily detected with an accuracy of 20-30 s. This event is also coincident with spindle elongation which is easy to monitor with a mCherry::Tubulin probe.

We are aware that we have made the shortcut between time in mitosis and the reduced ability of mutants to degrade Cyclin B. We now present a new supplementary Figure S1, showing measurements of the length of mitosis combined with the time of Cyclin B reporter degradation, for *polo* and *polo,mad2^P^* mutants. We now unambiguously correlate that a defective Cyclin B degradation is associated with a SAC-independent function of the Polo kinase.

Note that the Cyclin-B::GFP available transgenes are poorly expressed in neuroblasts and notoriously not suitable to determine accurately the mitotic entry point for mitotic studies.

3.- Expression of the constitutively active PoloT182D mutant was shown to restore the length of mitosis for the polo9/10 mutant by more than half, while a wild type version of Polo fully restored the normal time of mitosis (Figure 2D). The authors discarded the possibility that increased Polo activity could be detrimental for spindle assembly and triggered a SAC-dependent mitotic delay, instead proposing that the delay resulted by the excess of Polo activity in a SAC-independent manner. However, the experiment that supported this claim (measuring time in mitosis upon PoloT182D expression in mad2P and polo9/10 mad2P mutants, Figure 2E) does not rule out that the delay could be associated to spindle-related problems that do not necessarily affect kinetochore-microtubule attachments and thus do not activate the SAC.

Indeed, we agree that this is one possible mechanism that can contribute to our observed SAC-independent mitotic delays, which is not mutually exclusive with the mechanisms we already proposed. We have mentioned this hypothesis in our revised discussion. We are aware that high Polo^T182D^ expression creates kinetochore attachments defects, as well as a delayed mitotic progression. Barbosa and colleagues (EMBO, 2020) have showed this recently in a nice study, as well as other groups cited in this manuscript. These defects have already been characterized in details and while we understand that this is an important aspect of Polo function, the aim of our study was to not overlap with this publish work and focus on the time in mitosis. The point of using *mad2^P^* mutant was to get rid of the SAC-dependent mitotic delay caused by KT/MT attachments defects.

This highlights again the necessity for additional cell cycle markers other than mCherry-Tubulin.

Cyclin-B dynamics analyses were performed in the above-mentioned new Figure S1 (see also our responses to point 2)

Additionally, why the excess of Polo kinase activity in the PoloT182D mutant does not delay mitosis to the same extent in an otherwise wild type strain than in the polo9/10 background?

This is a good point. In our Western blot analyses, we showed that Polo^T182D^-GFP (and Polo-GFP) levels are low compared to the levels of endogenous Polo kinase present in WT brain extracts (See Figure 2 A). Therefore, we hypothesize that the dominant effect of the active Polo^T182D^-GFP variant is modest. These data are in agreement with the result of Barbosa et al., (EMBO, 2020): their Western Blot analyses showed that they o overexpressed much more of the untagged Polo^T182D^ compare to the tagged version we used. Congruently their overexpression experiments in WT flies causes a more substantial mitotic delay.

4.- Combination of ial and aurA mutations triggered a synergistic mitotic delay (Figure S2B-C). Did the authors check whether the introduction of the PoloT182D mutation in the aurA ial1689/ial2A43 background rescued this delay?

We performed this experiment recently. There is actually no rescue of the time in mitosis (see the modified Figure S3 C). This result suggests that in addition to Polo activation, one or more Aurora (A and B) substrates are required to control efficient Cyclin B degradation. A model for Aurora kinase dependent control of Polo T182 phosphorylation is now shown in a new Figure 6.

Finally, some minor concerns are: 

1.- Does the PoloT182A mutant generate any additive effects when introduced in the aurA or ial mutant backgrounds?

The Polo^T182A^ variant does not appear to generate stronger centrosomal, polarity and spindle orientation defects when expressed in *aurA* null mutant (new Figure 5 B, D, H , J). Expression of this variant increased the mitotic delay of *aurA* but not *ial* mutants (See new Figure 3).

2- Inactivation of the SAC and the associated increase in aneuploidy was shown to correlate with the severely enhanced tumor growth of weak polo mutant NSCs, while SAC inactivation in the strong polo hypomorph, despite similar aneuploidy rates, did not expand NSC number. However, the strong polo mutant already displayed high percentages of host flies developing tumors per se. It would be informative to know whether tumors in the polo9/10 mad2P background develop faster and are more aggressive. 

We actually tried to perform this experiment but we were not able to detect any differences in tumor development between *polo9/10* and *polo9/10,mad2^P^* mutants. This is the main reason why we also performed this experiment using weaker *polo* mutants.

Reviewer #2 (Significance (Required)): 

Gallaud et al. propose that Aurora A and B kinases modulate the activity of Polo kinase to coordinate progression through mitosis with polarization during the asymmetric cell division of Drosophila neural stem cells. The conclusions drawn in their manuscript could be of potential interest and represent a conceptual advance in the field, although as stated in Section A some of the authors claims need further experimental support. My main issue is that the estimation of the length of mitosis always relies on the analysis of spindle dynamics by live imaging of cells expressing fluorescently tagged tubulin. Based on the known roles of Aurora and Polo kinases on centrosome duplication and spindle dynamics, the authors should have included additional cell cycle markers to strengthen their conclusions.

As we explained above, we believe that mCherry-Tubulin is the most appropriate marker to monitor the time between NEB and Anaphase, and we do not see how centrosome or spindle defects in *polo* or *aurora* mutants would invalidate the use of mCherry-Tubulin to monitor NEB since it is based on visualization of the pool of free (unpolymerized) mCherry-Tubulin in the nuclear space.

The results from this manuscript are of interest for a broad readership but would be of special relevance to researchers studying the mechanisms that control asymmetric cell division, stem cell duplication and neurogenesis, as well as to those working in diseases that arise as a consequence of the deregulation of these processes. 

Regarding my expertise, I am a researcher working in the field of cell cycle control and interested in mitotic checkpoints, chromosome segregation and polarity establishment during cell division. 


Reviewer #3 (Evidence, reproducibility and clarity (Required)): 

The authors show that weak polo hypomorphs show a slight mitotic delay that is not affected by loss of Mad2 and strong hypomorphs the delay is longer and loss of Mad2 has some effect on decreasing time in mitosis. Similar checkpoint effects are seen in Aurora A mutants leading authors to ask whether Aurora kinases were activating Polo. They go on to show that a constitutively active form of Polo kinase is able to reduce the time that both Aurora A and Aurora B mutants spend in mitosis. Polo kinase activation at the centromere was assessed by monitoring anti-P-T182 and centrosomin at centrosomes. Together the data suggest that both Aurora A and B can phosphorylate and activate Polo. Brain transplantation experiments then show similar consequences wit polo mutants to those previously reported with Aurora A mutants. Moreover, constitutively active Polo was able to diminish the amplification of neural stem cells in Aurora A mutants and suppress the tumor formation by transplanted Aurora A mutant brains, a phenoptye reflected by the suppression of cell polarity markers. An excellent study. 

Reviewer #3 (Significance (Required)): 

I strongly recommend publication of this paper without the need for revision. The experiments have been carefully carried out and thoughtfully interpreted such that the conclusions are fully justified. The finding that Aurora kinases phosphorylate and activate the major Polo mitotic kinase in regulating the polar fate determining divisions of fly neuroblasts is a finding of profound importance.

We share this reviewer’s enthusiasm.
